# On the High Sensitivity of the Electronic States of 1 nm Gold Particles to Pretreatments and Modifiers

**DOI:** 10.3390/molecules21040432

**Published:** 2016-03-31

**Authors:** Oxana Martynyuk, Yulia Kotolevich, Rodrigo Vélez, Jesus Efren Cabrera Ortega, Hugo Tiznado, Trino Zepeda Partida, Josué D. Mota-Morales, Alexey Pestryakov, Nina Bogdanchikova

**Affiliations:** 1 Centro de Nanociencias y Nanotecnología, Universidad Nacional Autónoma de México (UNAM), Ensenada 22860, Mexico; martynyuk_oksana@mail.ru (O.M.); Julia.Kotolevich@gmail.com (Y.K.); rovera91@hotmail.com (R.V.); tiznado@cnyn.unam.mx (H.T.); trino@cnyn.unam.mx (T.Z.P.); 2 Department of Physical and Analytical Chemistry, Tomsk Polytechnic University, Tomsk 634050, Russia; pestryakov2005@yandex.ru; 3 Centro de Investigación Científica y de Educación Superior de Ensenada, Ensenada 22860, Mexico; efren507.507@gmail.com; 4 CONACYT Research Fellow at Centro de Nanociencias y Nanotecnología, Universidad Nacional Autónoma de México (UNAM), Ensenada, 22860, Mexico; jmota@cnyn.unam.mx

**Keywords:** FTIR CO, electron stage of gold, 1 nm gold, sensitivity to pretreatment

## Abstract

In this paper, the effect of modifiers and pretreatments on the electronic states of 1 nm gold nanoparticles (AuNPs) supported on silica was systematically studied. AuNPs deposited on silica (particle size of 2–4 nm) modified with Ce, La and Fe oxides, were studied by FTIR of adsorbed CO after different redox treatments at 100, 300 and 500 °C. This study was conducted at room temperature to allow detecting the electronic states of gold, which is more likely involved in CO oxidation at the same temperature. AuNP size distribution was measured by HRTEM. It is shown that the electronic state of gold species (Au_n_^δ−^, Au^0^, Au_n_^δ+^, Au^+^) in 1 nm AuNPs is sensitive to the modifier as well as to the temperatures of redox pretreatments. Supports modified with the same additives but containing larger AuNPs (~3, 4, 5, and 7 nm) were also studied. They showed that Au^0^ remains stable irrespective of additives and redox pretreatments, indicating no significant effect of such treatments on the electronic properties of larger AuNPs. Samples with a predominant AuNP size of 2 nm are an intermediate case between these two groups of materials.

## 1. Introduction

It is well accepted that in gold-based catalysts, the size of gold nanoparticles (AuNPs) plays a major role in catalytic activity. Thus, the smaller the AuNPs the better their performance when supported on titania, particularly in CO oxidation and water-gas shift (WGS) reactions [1,2,3,4].

The activity of fairly monodispersed AuNPs has been studied in detail only in the size interval from 5 to 1.8 nm. It is believed that the maximum activity of AuNPs smaller than 5 nm corresponds to nanoparticles with an average size of 2.5 nm [5,6,7,8,9,10,11,12]. Gold species of less than 1 nm which include gold ions [13,14,15,16,17,18] and sub-nanometer gold aggregates [19,20,21] have also been tested for their catalytic activity.

Much effort has been made to elucidate the size-performance relationship of AuNPs in catalysis, but to date a correlation that includes a wide range of sizes (from 1 atom to 5 nm) is still to be developed. The threshold at which activity has a maximum, if it exists, has not reached a general consensus yet.

In this regard, the Flytzani-Stephanopoulos group attempted to connect the abovementioned intervals (2–5 nm and <2 nm) for WGS reactions. They found that AuNPs deposited on Fe_2_O_3_, CeO_2_ and Al_2_O_3_ are inactive, while ions and sub-nanometer clusters, on the contrary, are characterized by high activity. Flytzany-Stephanopoulos and collaborators have shown that some AuNPs, representing up to 94%–97% of the total gold content, behave as spectators in the case of WGS. In the same line, both AuNPs and subnanometer Au clusters have shown to be active towards CO oxidation. Nevertheless, it is uncertain whether 1 nm Au NPs are active or not, taking into account that they are an intermediate case between the reported optimal size of 2.5 nm and Au clusters [16,22,23,24,25]. The main difficulty in dealing with particles of 1 nm is that obtaining such a size with a narrow size distribution is not an easy task, since these particles easily sinter unless they establish a strong interaction with a support.

It is important to note that in the study of particles of such a narrow size distribution, a variation of only 0.5 nm with a predominant particle size of 1 nm represents an error of 50%. In the literature the term monodispersity is frequently used even when the size corresponding to the maximum in the distribution histogram accounts for less than 50% of the total population. So, when measured, catalyst activity in fact includes contributions from all sizes and both active and inactive nanoparticles. Therefore, the most important task for the study of size-activity dependence of AuNPs is the synthesis of monodispersed nanoparticles of less than 2 nm, particularly in the gold case, where sintering of small nanoparticles and aggregates readily occurs.

Leaving aside the monodispersity issue, there are scant reports on the synthesis of supported AuNPs of 1 nm in size. We have found only two works dedicated to this synthesis on mesoporous silica. In the publication of Tsukuda *et al.*, 1 nm AuNPs were produced from an expensive precursor (Au_11_^3+^ magic clusters, protected by triphenylphosphine), besides of yielding a product with very low loading of active metal (0.07 wt % Au) [26,27]. Yang *et al.* also reported on the synthesis of 1 nm AuNPs using a complex support (mesoporous SiO_2_-coated graphene oxide nanosheets) under high pressure (~60 atm) of CO_2_ [28]. Those methods describing the preparation of supported monodisperse AuNPs of 1 nm size are sophisticated, expensive and laborious. Sometimes a narrow size distribution of 1 nm of gold is obtained by chance [29]. Such results are difficult to reproduce, so when analyzing the literature, this type of publications cannot be taken into account.

Infrared absorption (IR) spectroscopy has proven to be one of the most powerful spectroscopic techniques available for the characterization of catalytic systems [30,31,32,33,34,35]. Information extracted from IR data provides one of the most detailed sources of identification data of adsorbed intermediates and allows characterizing the surfaces of catalysts [36]. In IR studies the quantification of signal intensities of probe molecules also offers information about specific catalytic sites [37].

In this paper the synthesis of catalysts containing monodispersed 1 nm Au species supported on silica is reported. Metal oxide addition to supports was performed with the aim of modifying the catalyst performance. The samples were studied by FTIR of adsorbed CO under different redox treatments. This method is very sensitive to variations in the electronic state of supported metals [36] and this contribution sheds light on the sensitivity of 1 nm AuNPs towards redox treatment at different temperatures.

## 2. Results and Discussion

**Figure 1:**
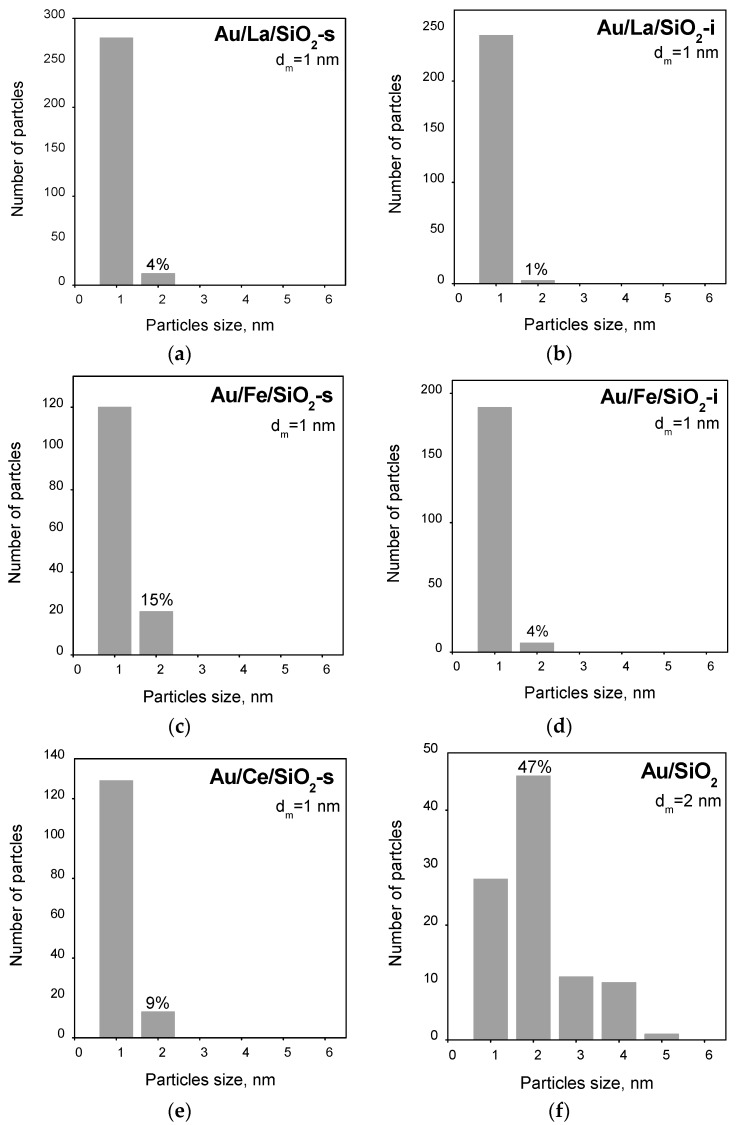
Au particle size distribution obtained by HRTEM: Au/La/SiO_2_-s (**a**); Au/La/SiO_2_-i (**b**); Au/Fe/SiO_2_-s (**c**); Au/Fe/SiO_2_-i (**d**); Au/Ce/SiO_2_-s (**e**); Au/SiO_2_ (**f**).

Figure 1 shows the size distribution of AuNPs on both unmodified silica and silica modified with metal oxide additives, e.g., La, Fe and Ce. It can be seen that the distributions of AuNPs on the modified supports (Figure 1a–e) are characterized by a high monodispersity with a predominant size of 1 nm. As it was reported in our earlier work [38], they were obtained by depositing gold on modified silica composed by SiO_2_ particles with very low size (2–4 nm). In Au/SiO_2_ (Figure 1f) the distribution of particles is wider, e.g., in the range of 1–5 nm with an average diameter of 2 nm. We also cannot exclude the existence of Au subnanometer species (undetectable by HRTEM) in the catalyst here studied.

**Figure 2:**
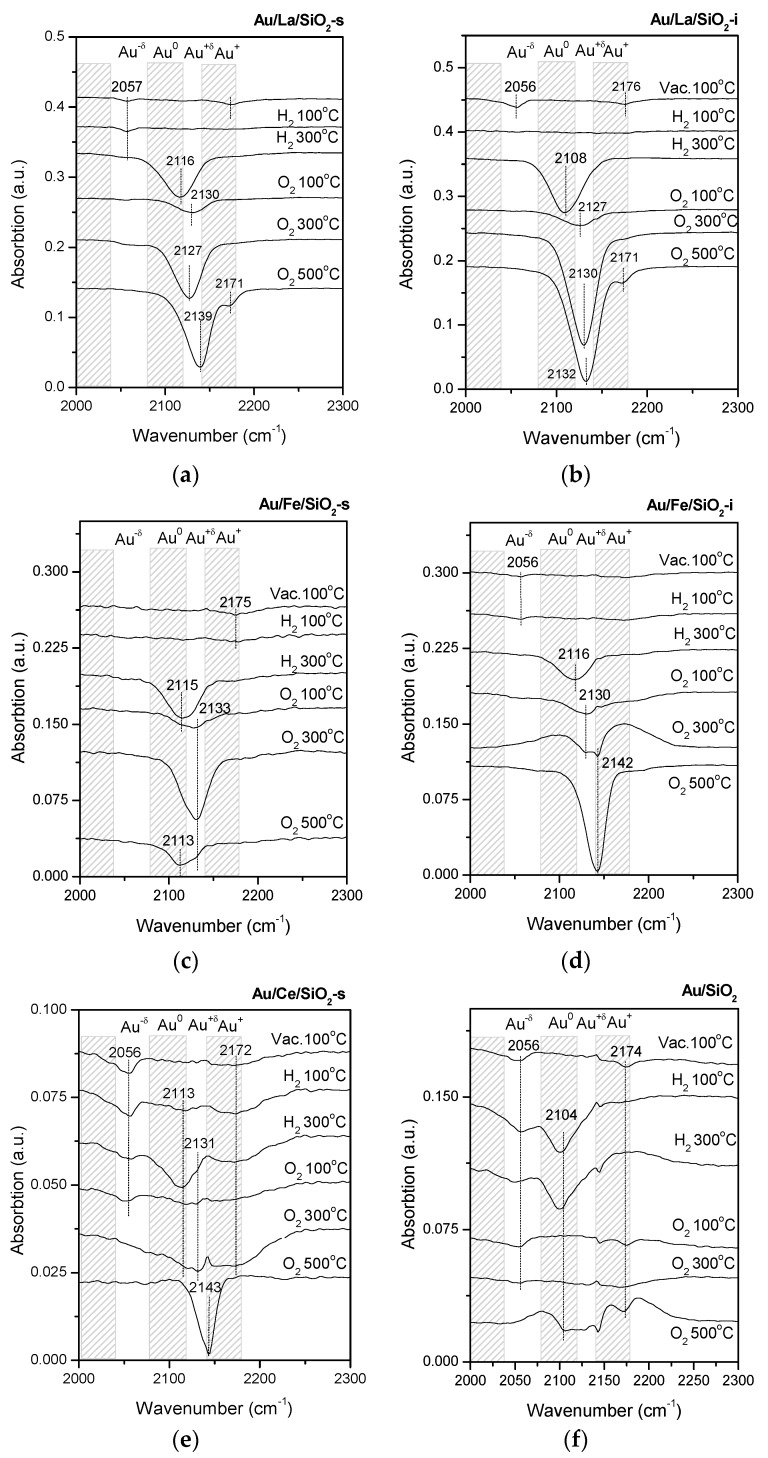
FTIR spectra of CO adsorbed on Au/La/SiO_2_-s (**a**); Au/La/SiO_2_-i (**b**); Au/Fe/SiO_2_-s (**c**); Au/Fe/SiO_2_-i (**d**); Au/Ce/SiO_2_-s (**e**); Au/ SiO_2_ (**f**) after different treatments.

In Figure 2 the FTIR spectra of adsorbed CO on Au catalysts with different modifiers and after different pretreatments are shown. The interpretation was made based on literature data [4,30,33,39,40,41,42,43,44,45] and on our earlier reports [19,46,47,48,49,50,51,52,53]. For most samples pretreated in vacuum at 100 °C, the absorption spectra observed in the range 2140–2180 cm^−1^ correspond to the adsorption of CO on Au^+^, and bands in the range of 2050–2080 cm^−1^ are interpreted as the adsorption of CO on Au_n_^δ−^ clusters and/or on relatively large AuNPs. Taking into account the histograms of Figure 1, the band in the range of 2050–2080 cm^−1^ could not correspond to large Au particles. Adsorption of CO on Au/Fe/SiO_2_-s, Au/Mg/SiO_2_-i and Au/Mg/SiO_2_-s after this pretreatment is not observed. It probably indicates the presence of gold in Au^3+^ state since it does not adsorb CO under the studied conditions [54].

Subsequent reduction in H_2_ at 100° C does not lead to significant changes in the electronic state of gold in the modified catalysts, however, reduction to the Au^0^ state (band at 2080–2120 cm^−1^) occurred in Au/SiO_2_. This may indicate weak metal-support interactions in Au/SiO_2_ when compared to the modified Au catalysts.

Pretreatment of all samples, in H_2_ at 300 °C, leads to the reduction of Au^3+^ ions and the appearance of Au^0^-CO signals (band at 2080–2120 cm^−1^). After subsequent oxidation at 100 and 300 °C, more oxidized forms of Au_n_^δ+^ and Au^+^ become dominant in all samples. In the case of Ce-modified and unmodified Au catalyst, no signals were observed. Again this may indicate that in all samples Au is oxidized to Au^3+^ (which does not adsorb CO under the studied conditions). Spectra of Au/Сe/SiO_2_-s and Au/SiO_2_ present signals in the absorption interval that corresponds to CO-Au^δ−^, and this species disappeared after oxidative treatment at 300 °C (due to the transition of this form to Au^3+^).

After pretreatment in O_2_ at 500 °С, deeper oxidation accompanied by the appearance of Au^+^ was observed in most of the samples. The exceptions were Au/Fe/SiO_2_-s and Au/SiO_2_, where carbonyls adsorbed on Au^0^ (along with oxidized Au species) were registered. Since reduction of Au_n_^δ+^ in an O_2_ atmosphere is not likely, this decomposition phenomenon could be related to decomposition of oxidized gold species at high temperature (500 °C). The observed instability of the oxidized gold species suggests weak interactions with the support (compared to the other samples). In turn, that correlates with the observed contribution of the Au NPs ≥2 nm. For samples presenting weak metal-support interactions (Au/SiO_2_ and Au/Fe/SiO_2_-s), this contribution is 70% and 15%, respectively, while for the other samples that showed stronger metal-support interactions, it is in the range of 1%–9% (Figure 1).

**Figure 3:**
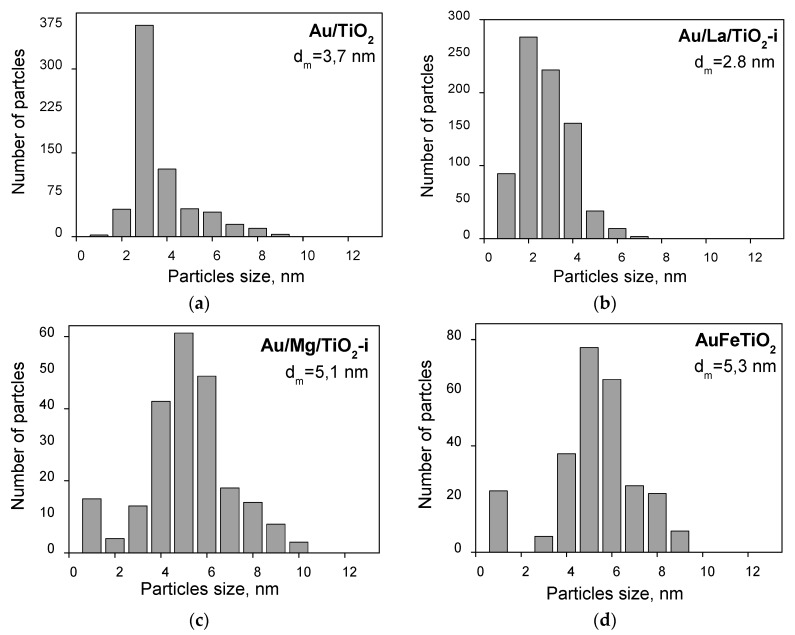

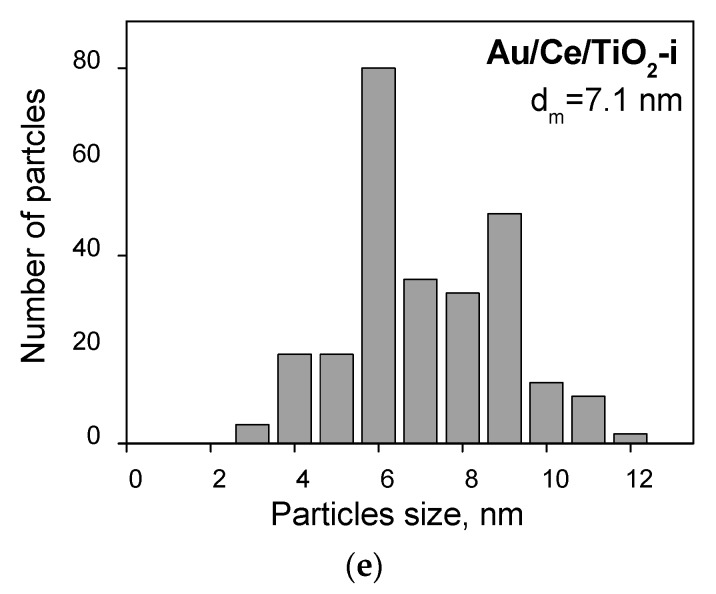
Au particle size distribution obtained by TEM for Au/TiO_2_ (**a**); Au/La/TiO_2_-i (**b**); Au/Mg/TiO_2_-i (**c**); Au/Fe/TiO_2_-i (**d**); Au/Ce/TiO_2_-i (**e**).

In order to study the effect of modification and conditions of pretreatments on the electronic state of AuNPs with size >2 nm, we used samples with AuNPs of average size of ~3, 4, 5, and 7 nm supported on TiO_2_ (Figure 3). For these Au catalysts, support modification was carried out using the same additives and in the same concentrations as those used for the previous set of samples by the impregnation method on silica.

**Figure 4:**
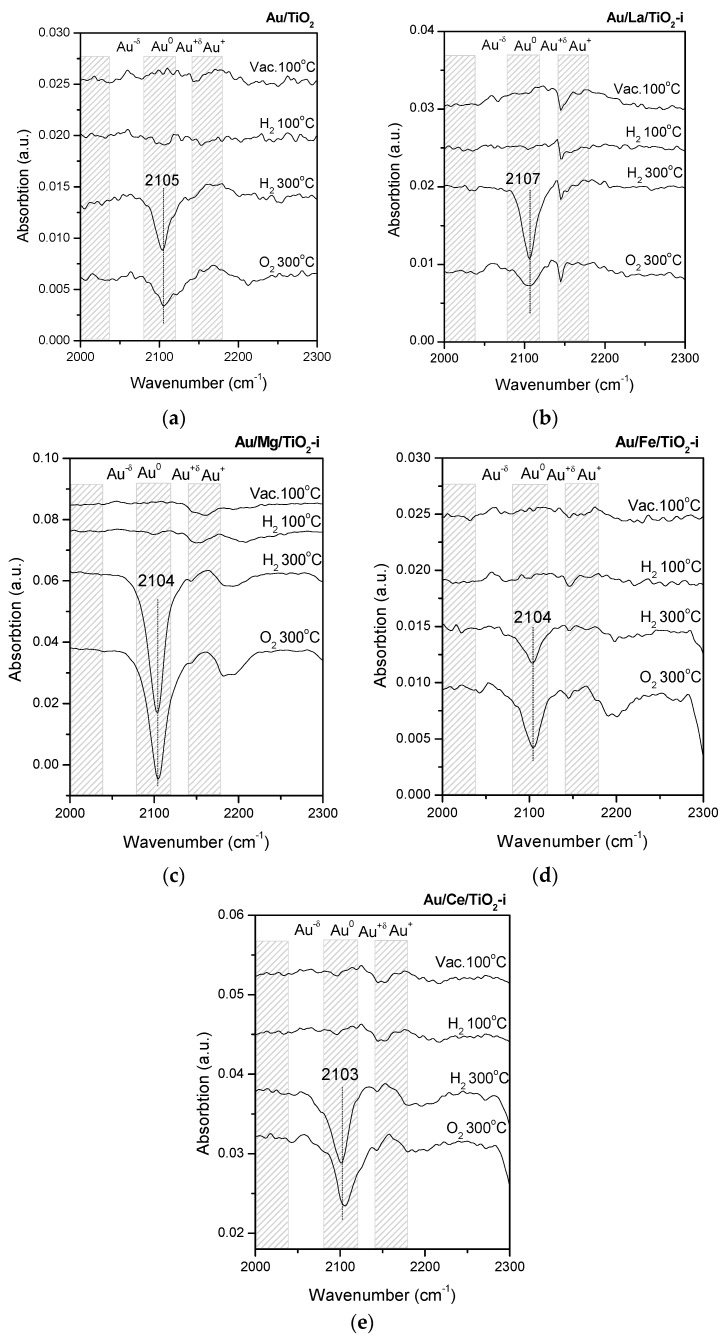
FTIR spectra of CO adsorbed on for Au/TiO_2_ (**a**); Au/La/TiO_2_-i (**b**); Au/Mg/TiO_2_-i (**c**); Au/Fe/TiO_2_-i (**d**); Au/Ce/TiO_2_-i (**e**).

As can be seen from Figure 4, for all pretreatment temperatures, all gas atmospheres (O_2_ and H_2_) and all modifiers, the formation of metallic gold state (CO-Au^0^, band at 2103–2107 cm^−1^) was the only phenomenon observed. This evidenced the lack of significant influence of all the studied additives and redox pretreatment conditions on the electronic properties of AuNPs with an average size from 3 to 7 nm. The intensity of CO absorption peaks in this series of samples compared with those of AuNPs ≤2 nm decreased by 4–10 times. Au content in samples with Au > 2 nm is 2 times higher than in samples with Au < 2 nm (4 and 2 wt %, respectively), hence, this difference is due to larger Au particle size, lower specific surface area (and consequently lower concentration of active sites for CO adsorption) in 3–7 nm Au samples compared to 1 nm Au materials (Table 1).

The nature of the support in both groups, *i.e.*, silica in the case of 1 nm Au NPs and titania in case of other sizes, may have implications for AuNPs’ susceptibility to treatments. This fact hints the possibility of titania buffering an eventual modification of the electronic state of AuNPs supported there, as it can also be reduced or oxidized during treatments. However, the electronic states of AuNPs on titania proved to be insensitive to treatments, regardless of the modification of the titania support with even more reactive oxides than the support itself, namely La, Ce and Fe oxides.

We did not find in the literature works dedicated to the study of 1 nm monodispersed supported AuNPs by FTIR of adsorbed CO. This is not surprising because there are only a few investigations devoted to the preparation of this type of particles [26,27,28]. In our previous publications, the results obtained by FTIR of adsorbed CO demonstrated the sensitivity of gold electronic states to redox pretreatments. It is important to underline that for all these works, the presence of Au subnanometer species was proved. Other works [19,49,50] showed the presence of CO-Au^δ−^, CO-Au^δ+^ and CO-Au^+^ absorption bands for Au species located inside mordenite channels with diameter of 0.65 nm after reduction pretreatment at 300 °C in CO; while a CO-Au^0^ band was not observed. This may indicate that, in the case of Au species with size ≤ 0.65 nm, no metallic structures are formed, while for 1 nm AuNPs metallic Au species can be formed, as shown in the present article. Also in our previous works with zeolites [48,49,50], and for SiO_2_ [53] with higher Au particle size (1–2 nm), a sensitivity to redox pretreatments was also observed. In these studies existence of subnanometer species was suggested.

In another work [52], the average Au particles size on SiO_2_ was 5.4 nm, according to TEM data, and by applying different methods the presence of CO-Au_n_ clusters was suggested. For these samples, bands ascribed to CO-Au^+^, CO-Au^0^ and CO-Au^δ+^, were observed by FTIR of adsorbed CO. In other publications [47,48], in Au/Al_2_O_3_ catalysts and those modified by Ce, Zr, La and Cs oxides, the Au particle size was 31–66 nm according to TEM and XRD data. Part of the support surface (20%–40%) was not covered by these large metal particles, and they contained highly-dispersed Au species identified by CO-Au^+^ and CO-Au^δ+^ bands in the CO FTIR spectra. In another work [46], Au/MgO characterized by 10%–18% of Au_n_^δ+^ small clusters and (Au)_n_ metallic Au particles, dispersed ionic species of gold formed after reduction at 350 °C played a crucial role in the activation of the CO triple bond. Moreover, the presence of “Au_m_^δ+^-Au_n_” ensemble sites were suggested.

Herein, on the basis of our analysis of literature data, the following tendency is presented: the sensitivity of adsorbed CO measured by FTIR to redox pretreatment is observed only for samples containing Au NPs of ~1 nm, registered or suggested on the basis of the study by combination of different methods. Nevertheless, more studies of Au NPs with different sizes are necessary. We also cannot exclude the existence of Au subnanometer species in the samples studied here.

Other papers investigated the sensitivity of the Au catalysts to pretreatment by FTIR of CO, where adsorption was carried out at low temperatures (−196 °C) [45,55,56,57]. It should be noted that under these conditions, weak adsorption of CO, including its physical adsorption on Au and supports, takes place. We conducted the study of CO adsorption at room temperature because this allows detecting the electronic states of gold, which are more likely involved in CO oxidation at that temperature.

## 3. Materials and Methods

### 3.1. Support Preparation

Mesoporous silica was synthesized by a neutral S^0^I^0^ templating route (S^0^—neutral primary amine surfactant; I^0^—neutral inorganic precursor) [58,59]. Dodecylamine was employed as surfactant and mesitylene as swelling organic agent [60]. The reaction products were filtered, washed with distilled water, and dried at room temperature for 24 h and at 100 °C for 2 h. The template was then removed by calcination, increasing the temperature at a rate of 2.5 °C min^−1^ and maintaining it at 550 °C for 3.5 h in air.

Incorporation of metal oxides (M) was carried out by two methods, namely S and I. Method S incorporates modifier precursors in the synthesis of SiO_2_ described above. Cerium (III) nitrate hexahydrate, iron (III) nitrate nonahydrate or lanthanum (III) nitrate hexahydrate were used as modifier precursors, with an atomic ratio Si/M = 40. The s-samples were filtered, washed with distilled water, and dried at room temperature for 48 h, followed by drying at 110 °C for 4 h, and then the samples were calcined at 550 °C for 4 h in static air. Method I was done by impregnation of the pure silica support using aqueous solution of M from the same metal precursors, 1.5 cm^3^/g, having the concentration needed to obtain a final molar ratio Si/M = 40. These i-samples were dried and calcined under the same conditions previously described for co-precipitation.

Degussa P25 titania (45 m^2^·g^−1^, nonporous, 70% anatase and 30% rutile, purity > 99.5%) was used as starting support. Before use, TiO_2_ was dried in air at 100 °C for at least 24 h. Modification of titania was made by impregnation (2.5 cm^3^/g) of initial TiO_2_ with aqueous solutions of the same precursors or magnesium nitrate with molar ratio Ti/M = 40. Then, impregnation products were dried at room temperature for 48 h and at 110 °C for 4 h, and calcined at 550 °C for 4 h.

### 3.2. Catalysts Preparation

For the preparation of nanoparticles in silica supports, gold complex, Au(en)_2_Cl_3_, was synthesized as described elsewhere [61]. Briefly, ethylenediamine (en, 0.45 mL) was slowly added to HAuCl_4_·3H_2_O (1.0 g) solution in deionized water (10 mL) and washed with ethanol (70 mL). The precipitate produced was filtered, washed with ethanol and dried at 40 °C under vacuum overnight. Au(en)_2_Cl_3_ (0.5 g) was dissolved in water (50 mL) adjusting the pH to 10.0. Silica support (modified or unmodified) (1.0 g) was added and stirred at 60–70 °C for 2 h. The reaction products were filtered, washed with distilled water and dried in vacuum at 70 °C for 5 h. Finally the materials were thermally treated at 300 °C in H_2_ for 1.5 h to ensure a complete removal of the organic template.

Commercial HAuCl_4_·3H_2_O (Aldrich, Saint Louis, MO, USA) was used as gold precursor. Au/TiO_2_ and Au/M/TiO_2_ catalysts (nominal loading 4 wt % Au,) were prepared by deposition–precipitation with urea in the absence of light, following the previously reported procedure [61,62,63]. Briefly, the gold precursor (4.2 × 10^−3^ M) and the urea (0.42 M) were dissolved in 50 mL of distilled water; the initial pH of the solution was 2.4. Then, 1 g of support was added to solution, the suspension temperature was increased to 80 °C and kept constant for 16 h under stirring. After the deposition–precipitation procedure, all samples were centrifuged, washed with distilled water four times, centrifuged again, and dried under vacuum for 2 h at 80 °C. After drying, the samples were stored at room temperature in a desiccator under vacuum, away from light, in order to prevent any alteration [63].

### 3.3. High Resolution Transmission Electronic Microscopy

High-resolution transmission electronic microscopy (HRTEM) studies were carried out using a JEM 2100F system (JEOL, Peabody, MA, USA) operating with a 200 kV accelerating voltage. The samples were ground into a fine powder and dispersed ultrasonically in isopropyl alcohol at room temperature. Then, a drop of the suspension was placed on a lacey carbon-coated Cu grid. At least ten representative images were taken for each sample. Particle size distribution was obtained by counting *ca*. 100 particles for each sample. The distribution of M and Au in the samples was obtained by means of the Z-contrast mode.

### 3.4. FTIR Spectra of Adsorbed CO

Fourier transformed infrared (FTIR) spectra of CO adsorbed on the sample surface were recorded in a Tensor 27 FTIR spectrometer (Bruker, Ettlingen, Germany) in transmittance mode with a resolution of 4 cm^−1^. *In situ* experiments were carried out in a glass cell with NaCl windows capable of working at temperatures from −100 to 500 °C and pressures from 10^−2^ to 760 Torr. The sample powder ~20 mg was pressed into self-supporting disks of 13 mm in diameter. The sample was pretreated in H_2_ or O_2_ (100 Torr) at 100, 300 or 500 °C for 1 h and then cooled down to room temperature. Pretreatment in O_2_ was used to model the influence of an oxidative atmosphere used in the catalytic reaction. After that, H_2_ or O_2_ was evacuated and CO adsorption (research grade, P^0^ = 30 Torr, Matheson, Basking Ridge, NJ, USA) was carried out. CO spectra presented in this work were obtained by subtracting the CO gas phase spectrum, previously recorded without sample, also at 30 Torr (blank).

## 4. Conclusions

In the present work, a systematic study of monodispersed gold nanoparticles with a size of 1 nm, deposited on silica with particle size of 2–4 nm, is reported. It is shown that the electronic state of gold (Au_n_^δ−^, Au^0^, Au_n_^δ+^, Au^+^) in such small particles is sensitive to the nature of the support surface as well as to temperature and atmosphere of any redox pretreatments.

Supports modified with the same additives with a larger average size of supported gold particles (~3, 4, 5, and 7 nm) were also studied. They demonstrate the stability of Au^0^ electronic state (band at 2103–2107 cm^−1^) for all studied additives and redox pretreatment conditions, indicating the absence of any significant effect of such treatments on electronic properties of larger particles. The sample with predominant Au particle size of 2 nm, is an intermediate case between these two groups of materials.

## Figures and Tables

**Table 1:** Gold content and surface area of all supports and catalyst.

Sample Content	Au wt %/M wt %	S_BET,_ cm/m^2^	
		Support	Catalyst
Au/La/TiO_2_	3.6/5	45	45
Au/Ce/TiO_2_	3.5/5	43	47
Au/Mg/TiO_2_	4.0/1	43	44
Au/Fe/TiO_2_	4.2/2	46	44
Au/TiO_2_	4.5/0	56	46
Au/La/SiO_2_-s	2.3/7	325	110
Au/La/SiO_2_-i	2.4/7	242	145
Au/Fe_x_O_y_/SiO_2_-s	2.8/3	422	334
Au/Fe_x_O_y_/SiO_2_-i	2.5/3	459	195
Au/CeO_2_/SiO_2_-s	2.7/7	271	271
Au/SiO_2_	2.5/0	345	228

## Abbreviations

The following abbreviations are used in this manuscript:

ac-HAADF/STEM	aberration-corrected annual dark field scanning transmission microscope
Au NPs	gold nanoparticles
HRTEM	high resolution transmission electron microscopy
WGS	water-gas shift reaction
TPR	temperature-programmed reduction
FTIR CO	Fourier transform infrared spectroscopy of adsorbed CO
MSI	metal-support interaction
